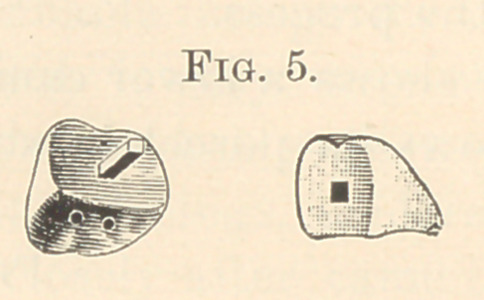# A System of Detachable Facings

**Published:** 1896-08

**Authors:** W. L. Mason

**Affiliations:** Red Bank, N. J.


### A SYSTEM OF DETACHABLE FACINGS.

BY W. L. MASON, D.D.S., RED BANK, N. J.

Mr. President and Gentlemen,—I present to you to-night, for your consideration, a system of detachable facings for crown- and bridge-work,—a system that does not in any way change the appearance of the perfectly-made crown or bridge of to-day.

About sixteen years ago porcelain facings, soldered to gold bands, were put in general use, and closely after came the bridge-work, very crude at that time, but advancing rapidly to the almost perfect construction of to-day. I could say perfect but for one fault, and that is the uncertainty of the condition of the porcelains after soldering and cementing into position. I know that we have teeth to-day that stand the strain better in the process of forming them into crowns or into bridges, but they will never be made to expand or contract like metal. We may be very careful in heating an invested piece for soldering, and cool off properly, and try the piece in, and have no strain upon it, so we cement in position. As near as we can see we have a perfect piece of bridge-work. But, just as long as the piece is in service, the porcelains are apt to separate from their backings. I think it is a great necessity that we attend to the future conditions which are apt to happen with a porcelain on crown or bridge. There is constant possibility of having a patient back with a porcelain broken off, and we should so construct our work as to replace damage quickly, not only to the patient's comfort, but also to our own.

I will acknowledge that I have never repaired a bridge in the mouth yet to my own satisfaction, where the porcelain was soldered to its backing. Is it practical to have a porcelain detachable? It must be, for scores have tried to produce them. Patent records show the efforts made. Up to the present time none have been invented so that they can be manufactured and sold to the dentist for his immediate use, and whether they are practical or not, I would leave for you to examine the mode of forming, and judge for yourselves.

In my judgment, a detachable porcelain is just as important to crown- and bridge-work as crown- and bridge-work is to dentistry. For a number of years I have been seeking a mode of constructing the porcelains so that they would be separate but have a perfect contact with the backings, and be equal to the facings now in use. Through that effort I have produced a system of dovetail and grooves to match, and a process of manufacturing whereby a porcelain is made independent of its backing, and a porcelain from one mould will fit each and every one of the backings made for that size mould, or universal in their use.

Before going further, I will call your attention to a few illustrations of the process.

Fig. 1 shows a lower canine crowned and cemented to its root. Fig. 2 shows its porcelain sliding from its backing. Fig. 3 shows

FIG. 1.

FIG. 2.

FIG. 3.

porcelain and backing separate. This illustrates the mode of constructing the anterior upper and lower six teeth. In Fig. 3 we have a metal dovetail fitted perfectly to the back of the porcelain, and extending a little beyond its cutting-point; it also shows its solid backing with a groove to receive the dovetail. It will not be necessary to go into the detail of the construction of the parts, but only that part of the work that is left for you to finish, as the porcelains with the metal dovetail attached and the grooved backing are manufactured. The tooth with its backing is fitted to the band, by grinding out where necessary. Then wax the gold backing to band, and after wax is hard, take hold of extended portion of dovetail and draw from backing. The crown is now ready to invest. Be careful to fill up the dovetail groove and let investment material come over cutting-point of backing, so as to keep out all solder.

Heat up solder and cool as quickly as you like. After removing from investment, see that the groove is thoroughly cleaned and dried; also dovetail on porcelain. Now take some chloro-percha, quite thick, fill up groove, and force porcelain in position. Saw off (don't cut) the extended portion of metal dovetail. Then finish as usual. For use as a dummy articulate to position and join parts with wax. The condition for the posterior teeth I have changed somewhat. Fig. 4 shows a molar dummy, with its cusp and porcelain together, having the same general appearance of molar dummy in general use, with the exception that the porcelain takes up more space on its palatal portion, making a saving of gold.

Fig. 4.

Fig. 5.

Fig. 5 shows the dummy parted, giving a view of the joining parts of a solid gold cusp, the upper buccal portion sloping upward and backward towards the ridge, and having on its face a square pin extending forward and upward. Fig. 5 also shows porcelain with square hole extending from surface just above the cusp portion, upward and outward to receive square pin fitted to cusp. After placing cusp and porcelain together the dummy is ready to grind in position. Wax parts together, remove porcelain, and solder. Cement porcelain to pin, and finish as usual. The advantages gained by this method are many, and can only be appreciated by practice.

The first advantage will be that you do not have to place your teeth under the flame of a blow-pipe.

*Second.* You have a solid backing without bubbles, as all parts are dropped forged.

*Third.* You can heat up your invested piece quickly, and not have to take the usual care; also cool off quickly.

*Fourth.* The small amount of solder you have to use,—just enough to join parts together.

*Fifth.* Saving your porcelain from being etched by borax.

*Sixth.* You are able to fit a bridge, releasing the strain by cutting and resoldering, and not have the porcelain interfered with.

*Seventh.* The most important of all. The amount of time saved to the busy dentist will equal about half of the time spent in the old method, and being free from annoyance in spending part or whole of a day repairing a bridge, with this system the repair is but a matter of a few minutes. If you put a tooth of mould No. 22 on, and it should break, you may order an exact duplicate of same and slip it in position, keeping yourself in good humor, and giving your patient the greatest amount of satisfaction.

## Figures and Tables

**Fig. 1. f1:**
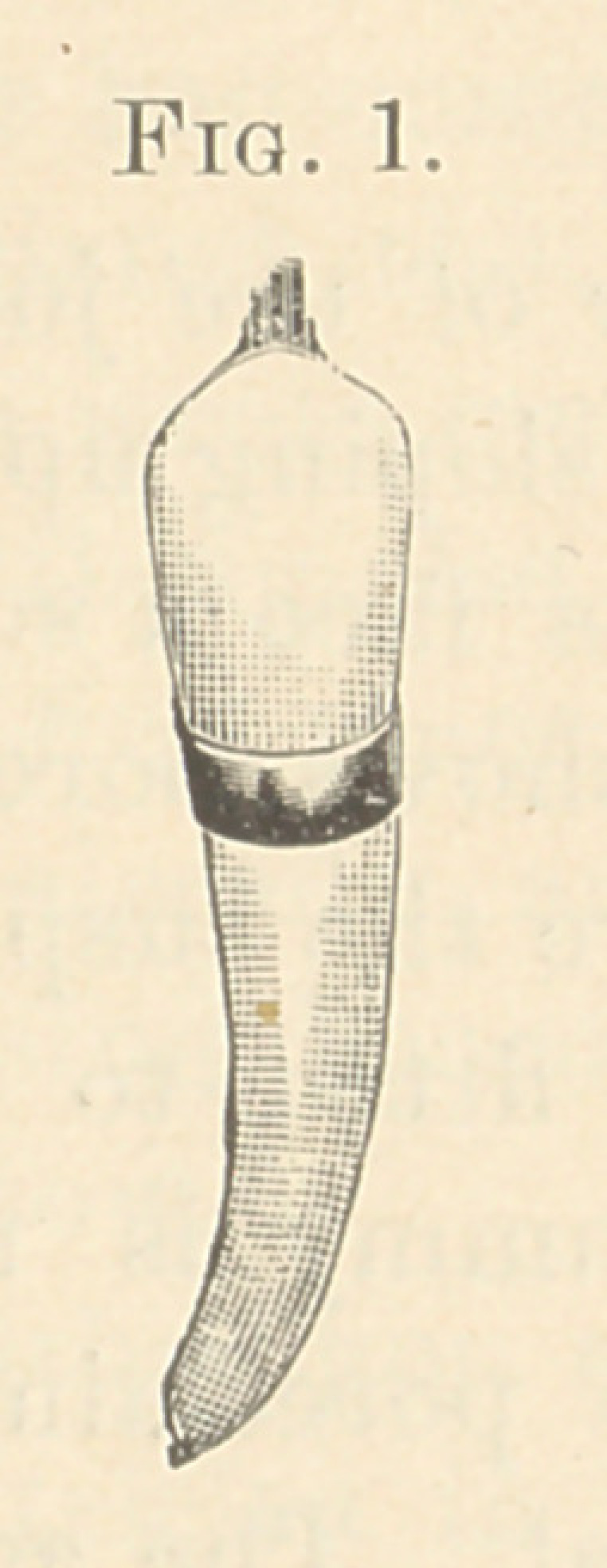


**Fig. 2. f2:**
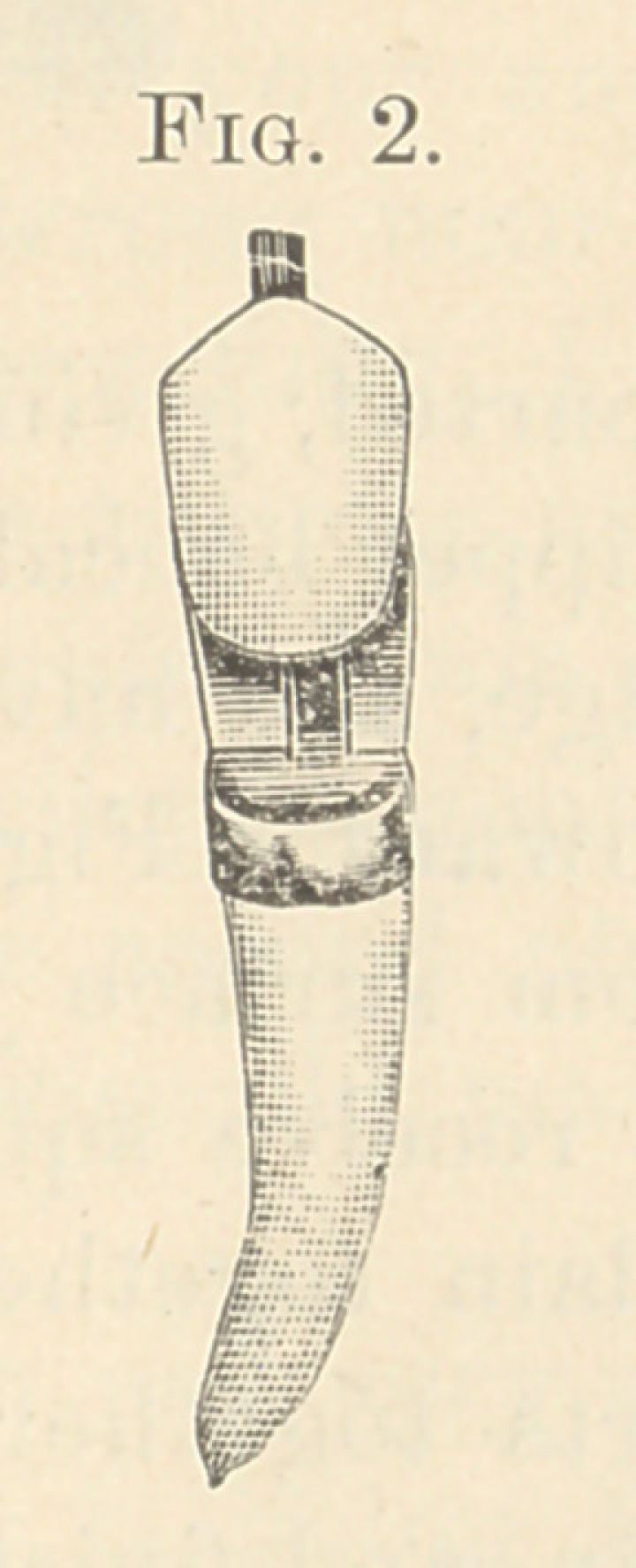


**Fig. 3. f3:**
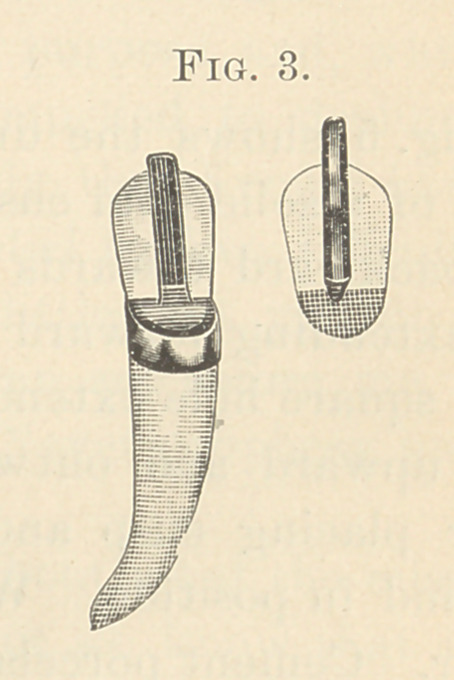


**Fig. 4. f4:**
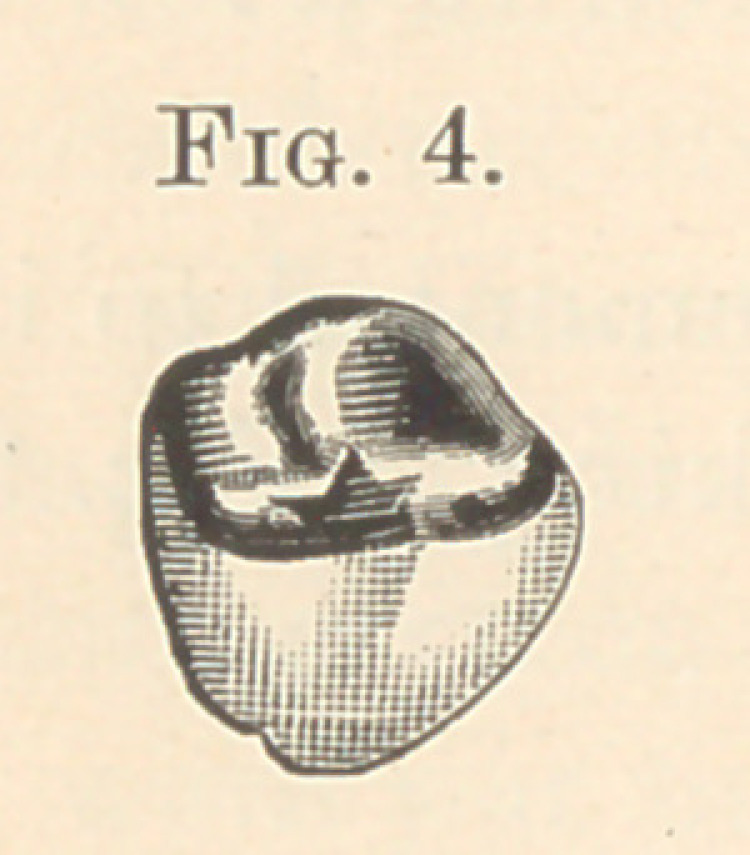


**Fig. 5. f5:**